# Coupling coordination and regional health equity: an empirical study of economic, social, and healthcare systems in Zhejiang Province, China

**DOI:** 10.3389/fpubh.2025.1581834

**Published:** 2025-06-18

**Authors:** Xiuwen He, Kai Lin

**Affiliations:** ^1^Public Health College, Hangzhou Normal University, Hangzhou, China; ^2^Zhejiang Evaluation Centre for Medical Service and Administration, Hangzhou, China

**Keywords:** health equity, health services accessibility, fuzzy-set qualitative comparative analysis, coupling coordination, regional health

## Abstract

**Introduction:**

Health equity remains a critical issue in regional development, particularly in areas with imbalanced economic, social, and healthcare resources. This study investigates the coordination among economic, social, and healthcare systems in Zhejiang Province, China, with a specific focus on promoting regional health equity.

**Methods:**

Data were collected from 86 counties in Zhejiang Province over a three-year period (2020-2022). A coupling coordination degree (CCD) model was employed to quantitatively assess the level of integration among the three systems—economic, social, and healthcare.

**Results:**

The analysis reveals that counties with robust social assistance mechanisms and accessible healthcare services consistently demonstrate higher CCD values, indicating better system coordination. In contrast, economically underdeveloped regions struggle to achieve synergy among the systems. Notably, persistent disparities are observed in mountainous and island counties, where coordination remains weak despite overall improvements across the province.

**Discussion:**

The findings underscore the importance of targeted policy interventions to bridge regional disparities. Integrating social welfare programs with healthcare infrastructure is essential to enhance system coordination and promote equitable access. These insights offer practical guidance for policymakers seeking to address healthcare inequality and support balanced regional development.

## 1 Introduction

The equitable allocation of healthcare resources is a fundamental pillar of high-quality healthcare system development, serving as a critical marker of societal modernization (1). At the same time, the promotion of health equity has emerged as a crucial driver of systemic coordination, expanding both the breadth and depth of universal health coverage (UHC) (2). Ensuring fair and inclusive access to healthcare services forms the essential foundation for achieving UHC, safeguarding basic health rights, and improving overall public health. Empirical evidence indicates that rational distribution of healthcare resources, such as reducing avoidable hospitalizations for chronic conditions like diabetes, directly enhances population health outcomes and reduces systemic healthcare burdens (3).

Increasingly, scholars recognize that the relationship between inclusive economic growth, public service equalization, and healthcare system development is mutually reinforcing. Inclusive economic growth facilitates broader sharing of development benefits and provides material conditions conducive to balanced public service delivery, including healthcare (4). Optimizing the allocation of economic resources helps narrow urban–rural disparities and strengthens healthcare accessibility (5). Public service equalization is not only a driver of inclusive growth but also a prerequisite for the high-quality evolution of healthcare systems, as it enhances human capital and reduces socio-economic vulnerabilities (6).

Meanwhile, the rapid economic expansion witnessed in regions like China has been accompanied by a surge in demand for healthcare services, posing challenges to achieving equitable and coordinated system development (7). Despite improvements in healthcare investment and accessibility, regional disparities in healthcare quality and resource distribution remain persistent. Health inequalities contribute to higher disease burdens among disadvantaged populations (8, 9), deepen social stratification, and diminish the benefits of economic growth (10).

Against this backdrop, enhancing the coordinated development of economic, social, and healthcare systems has emerged as a critical objective for sustainable and equitable regional development. Achieving such coordination requires not only investments in economic infrastructure and healthcare facilities but also systematic efforts to promote public service equalization, particularly in geographically disadvantaged areas such as mountainous and island regions.

To systematically assess the interaction between these subsystems, this study introduces a framework based on the concept of system coupling from physics, employing the Coupling Coordination Degree Model (CCDM) to measure the extent to which economic, social, and healthcare systems develop harmoniously or exhibit mutual constraints. However, recognizing that traditional quantitative approaches may oversimplify the complex causal relationships among multiple factors, the study further incorporates fuzzy-set Qualitative Comparative Analysis (fsQCA) to capture the multi-path configurations that drive coordinated development outcomes.

**Figure 1:**
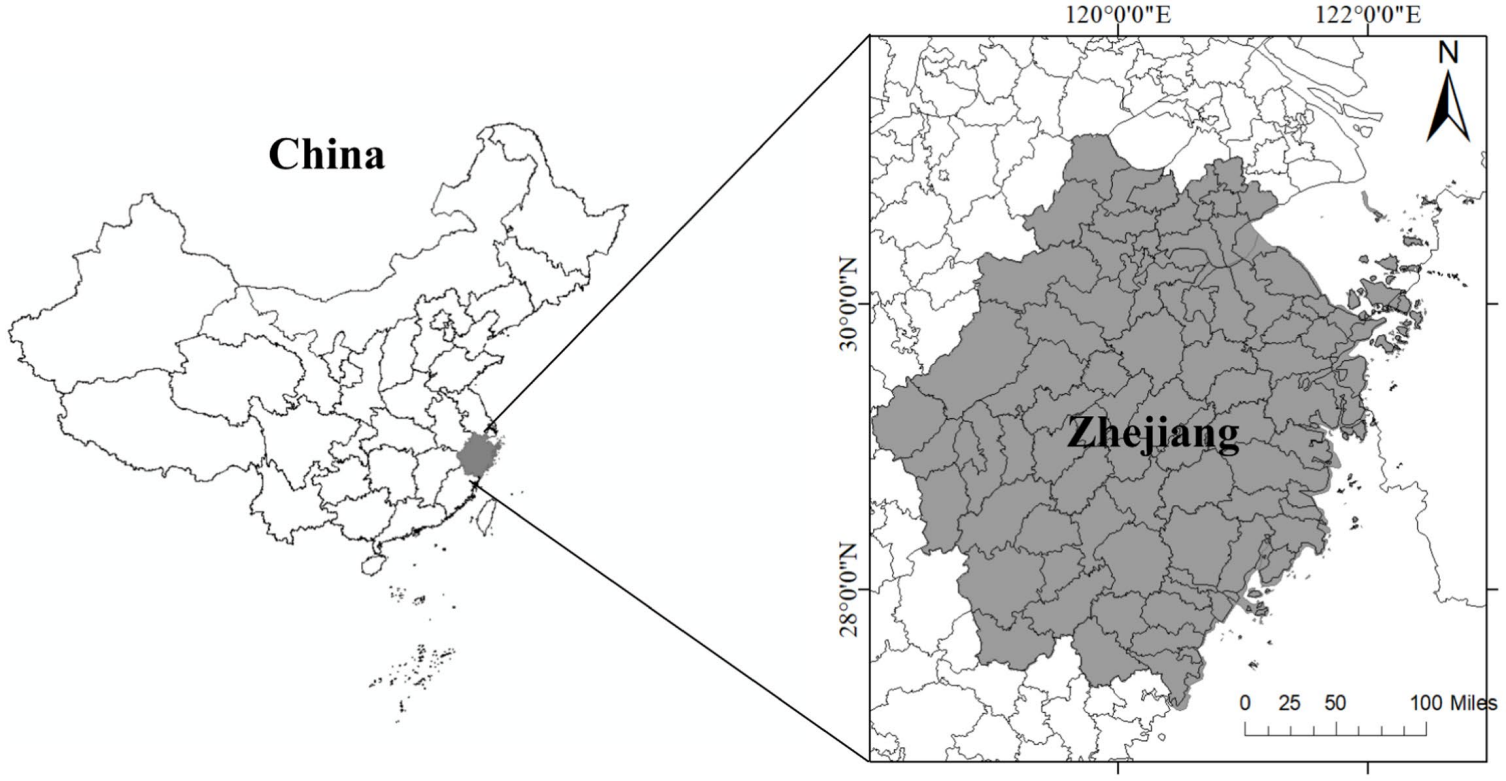
Study are of the Zhejiang in China.

Zhejiang Province, located in eastern China (Figure 1), offers a particularly relevant case for this investigation. As a pioneer in economic reform and institutional innovation, Zhejiang has achieved high levels of development but also faces significant internal disparities between urban cores and peripheral regions. In 2021, Zhejiang was selected as a pilot province for China’s “Common Prosperity” initiative, which places strong emphasis on public service equalization and healthcare system strengthening (11). Notably, the Mountain-Island Cooperation Initiative has been implemented to promote socio-economic integration between developed urban areas and less-developed mountainous and island counties. Focusing on county-level data from 2020 to 2022, this study examines how regional economic growth, social assistance programs, and healthcare accessibility interact to influence the degree of coupling coordination. The county level provides an ideal analytical scale because counties possess relatively complete socio-economic structures, enabling more nuanced examination of localized disparities and policy interventions.

Research Objectives and Strategies Specifically, this study aims to: (1) Measure the coupling coordination degree between economic development, social service provision, and healthcare accessibility across counties in Zhejiang Province, identifying patterns of spatial inequality and temporal change. (2) Investigate the causal configurations leading to high or low system coordination by applying a multi-configurational analytical approach combining CCDM and fsQCA, thus capturing the complex, non-linear drivers behind regional disparities. (3) To evaluate the role of public service equalization policies in promoting system coordination, and to provide policy-relevant recommendations for achieving regional health equity and inclusive socio-economic development.

## 2 Literature review

Existing research has extensively explored the relationship between economic development and healthcare resource allocation, mphasizing how economic growth can facilitate improvements in healthcare accessibility and service provision. Quantitative methods, such as the CCDM (12), the Theil index (13), and the Gini coefficient (14), have been widely employed to measure the coordination between economic growth and healthcare resource distribution. These studies have contributed significantly to identifying regional inequalities and demonstrating that economic prosperity generally promotes better healthcare access.

Building upon this foundation, recent studies have incorporated spatiotemporal analyses to capture the evolving patterns of healthcare distribution. For instance, Gong et al. and Wan et al. (15, 16) analyzed the dynamic characteristics of healthcare accessibility over time, revealing that although overall availability has improved, substantial regional disparities persist, particularly between urban and rural areas. These findings underscore that economic growth alone is insufficient to ensure equitable healthcare distribution, especially in regions facing geographical and infrastructural constraints.

In addition to macro-level assessments, a number of studies have examined the influence of specific factors such as social welfare programs, government interventions, and public health investments on the coordination between economic development and healthcare accessibility (17, 18). These works highlight the mediating role of social policies in linking economic growth with health equity. Nevertheless, such studies predominantly focus on isolated dimensions and often fail to capture the multisystemic interdependencies among economic, social, and healthcare structures.

Despite these advancements, several critical gaps remain in the existing literature. First, most studies tend to analyze economic development and healthcare resource allocation as independent or dyadic relationships, neglecting the complex and multidimensional interactions that exist when social assistance, public service equalization, and healthcare system development are considered together. Second, traditional approaches, predominantly based on regression models or static indices, often assume linear and symmetric effects between variables, overlooking the possibility of multiple causal configurations and nonlinear dynamics that are more consistent with real-world regional development processes (19, 20). Third, although spatiotemporal patterns have been analyzed, there remains a lack of fine-grained county-level investigations that can uncover more nuanced patterns of regional healthcare coordination, particularly within the framework of recent policy initiatives such as China’s Common Prosperity strategy (21).

Addressing these gaps, this study contributes in the following ways: (1) By incorporating public service equalization, it examines the joint effects of economic, social, and healthcare systems on coordination and coupling. (2) It introduces a multi-configurational analysis perspective to investigate causal mechanisms under different configurations, using qualitative comparative analysis (QCA) to explore driving factors and antecedent configurations for coordinated development between local public health services and economic growth. This study compares and explains the roles of these configurations in high-coordination development. (3) Through county-level analysis, it enriches research on the synchronized development of healthcare resource allocation and socio-economic growth in China, providing valuable references for middle-and high-income countries in terms of healthcare resource distribution.

## 3 Materials and methods

### 3.1 Data collection

Health inequalities result from a complex interplay of social, economic, environmental, and systemic factors (22). In selecting data indicators for the fsQCA analytical framework, the **SMART** principle (Specific, Measurable, Achievable, Relevant, Time-bound) is applied to ensure rigor and reliability. **Specificity** is ensured by selecting indicators directly related to the coupling coordination of economic, social, and healthcare (ESH) systems, such as public investment, healthcare accessibility, and social assistance. **Measurability** is achieved by using quantifiable metrics like healthcare facility density and social welfare coverage, enabling comparative analysis across regions. **Achievability** is addressed by ensuring data availability and consistency, using reliable sources such as national statistics, government reports, and regional development plans. **Relevance** is maintained by aligning indicators with international standards, including the number of assistant doctors and registered nurses per 1,000 residents. Lastly, **time-bound** selection ensures that data covers an adequate period (e.g., 2020–2022) to capture trends and policy impacts. By following the SMART principle, the selected indicators provide a solid foundation for fsQCA, facilitating a comprehensive evaluation of factors driving regional coordination in the ESH system.

Internationally, increasing attention has been devoted to the role of social assistance and healthcare service fairness in promoting health equity (23). Social assistance is generally defined as public programs aimed at providing economic support to disadvantaged populations to reduce poverty and vulnerability (24). It directly influences social determinants of health by ensuring basic living standards and mitigating health risks associated with socioeconomic deprivation. Similarly, healthcare service fairness refers to the equitable distribution of healthcare resources and services across different population groups and geographic areas, emphasizing both accessibility and affordability (24, 25). Common international metrics for evaluating social assistance include the proportion of the population covered by social protection programs, per capita expenditures on social welfare, and the adequacy of benefit levels (26, 27). For healthcare service fairness, metrics such as physician density, hospital bed density per capita (28), total health expenditure as a proportion of GDP (29) are widely recognized.

To assess healthcare service fairness, this study utilized indicators such as the number of healthcare professionals per 1,000 residents and the number of medical institutions, which are generally aligned with internationally accepted metrics. However, due to constraints in data availability at the county and district levels in Zhejiang Province, comprehensive international standard indicators could not be fully accessed. As a result, proxy indicators were selected based on their strong conceptual and empirical alignment with the constructs of interest. For instance, the number of urban residents enrolled in the minimum living security program was used to represent social assistance. These proxies maintain a high degree of theoretical relevance and ensure consistency with internationally recognized frameworks for evaluating health equity, despite the presence of data limitations (30, 31). Other dimensions were similarly mapped to theoretical constructs of health equity. Economic development indicators (e.g., GDP per capita) capture material living conditions that influence health outcomes, while social development indicators (e.g., the number of urban and rural residents participating in basic endowment insurance and the urbanization rate) reflect broader social determinants that shape opportunities for health across populations. In summary, each selected indicator not only fulfills the SMART criteria but also embeds a direct conceptual linkage to internationally recognized health equity frameworks, ensuring that the evaluation of coupling coordination robustly reflects the multifactorial determinants of regional health disparities. Based on expert discussions and data availability, the indicators for evaluating these systems, presented in Table 1, closely align with the CCD theory by representing the core attributes of each subsystem. These indicators support Zhejiang Province’s strategic initiatives, such as the *Mountain-Island Cooperation* and the *Demonstration Zone for Common Prosperity*. The Common Prosperity strategy is designed to promote coordinated development between urban and rural areas, improve the accessibility and quality of public services, and enhance overall societal well-being. As a concrete policy initiative within this broader framework, the Mountain-Island Cooperation program focuses on economically underdeveloped and geographically remote areas, particularly mountainous and island regions with low population density and limited access to urban infrastructure and services. Its primary objective is to stimulate regional economic growth while enhancing equity in the distribution of social welfare. To evaluate the effectiveness of this initiative in narrowing regional disparities and promoting public service equalization, the study employs key indicators such as social security coverage and healthcare service accessibility. These indicators provide an evidence-based means to assess the extent to which the initiative contributes to reducing spatial inequalities and advancing the goals of inclusive and balanced regional development.

**Table 1:** Specific indexes.

System	Subsystem	Indicator & indicator type	Unit	Description	Reference
Economy	Economic Aggregate (Z1)	GDP [x1]	100 Million Yuan	Gross Domestic Product	
		Fiscal Revenue [x2]	100 Million Yuan	Local Government’s Financial Status	
	Economic Structure (Z2)	Industrial Proportion in GDP [x3]	%	Distribution of Industrial Structure	
		Proportion of Tertiary Industry in GDP [x4]	%	Distribution of Industrial Structure	
	Economic Efficiency (Z3)	Per-capita GDP [x5]	Yuan	Comprehensive Economic Strength	
		Per-capita Disposable Income of Urban Residents [x6]	Yuan	Urban Resident Economic Conditions	
		Per-capita Disposable Income of Rural Residents [x7]	Yuan	Rural Resident Economic Conditions	
Society	Public Investment (Z4)	General Public Service Expenditure [x8]	100 Million Yuan	General Public Service	
		General Public Budget Expenditure [x9]	100 Million Yuan	General Education Expenditure	
	Social Security (Z5)	Number of Urban and Rural Residents Participating in Basic Endowment Insurance[x10]	10,000 Persons	Basic Endowment Insurance Coverage	
		Persons Participating in the Unemployment Insurance Program [x11]	10,000 Persons	Unemployment Insurance Coverage	
	Social Assistance (Z6)	Number of Urban Residents in Minimum Living Security Program [x12]	Persons	Social Assistance	
Healthcare	Accessibility (Z7)	Assistant Doctors per 1,000 Residents [x13]	Unit	Supply Capacity of Medical Services	
		Registered Nurses per 1,000 Residents [x14]	Unit	Supply Capacity of Medical Services	
		Number of Beds per 1,000 Residents [x15]	Unit	Supply Capacity of Medical Services	
		Urban and Rural Households Participating in the Basic Health Care Program [x16]	10,000 persons	Medical and Hygiene Guarantee	
		Birth Rate [x17]	‰	Birth Statistics	
	Fairness (Z8)	Number of Tertiary Hospitals [x18]	Unit	Hospital Coverage	
		Number of Township Hospitals [x19]	Unit	Community Health Services	
		Number of Village Clinics [x20]	Unit	Community Health Services	

### 3.2 Data sources

The economic, societal, and environmental data used in this study were sourced from the *Zhejiang Statistical Yearbook* (2020–2022). However, due to administrative division adjustments in *Hangzhou* in 2021, data for the districts of *Xihu*, *Gongshu*, and *Binjiang* were found to be deficient. These districts, among the most economically developed in Zhejiang Province, underwent significant restructuring, which likely disrupted statistical reporting continuity, resulting in missing or inconsistent data. Given their advanced economic status and potential to disproportionately influence the results, these four districts were excluded from the analysis to ensure accuracy and reliability.

This study centers on the Mountain-Island Cooperation, launched in 2021. Data from 2020, 2021, and 2022 were selected to assess the program’s impact before, during, and after its implementation. Based on economic development levels and geographic characteristics, the 90 counties in Zhejiang Province were categorized into two groups: *Mountainous/Island* counties (economically less active regions, such as *Yunhe*, *Jingning*, and *Qingyuan*) and Other counties (economically developed and moderately developed regions, including *Xiaoshan*, *Yuhang*, and *Beilun*). Data from healthcare institutions, including hospitals, primary health institutions, professional public health organizations, and other related facilities, were incorporated to evaluate healthcare equity across these diverse regions.

### 3.3 Framework for the evaluation method

This article employs the EM, CCDM, and fsQCA methods to construct a comprehensive evaluation framework. Determining the index weights is a crucial step in this process, as it reflects the influence of various factors in the evaluation. In this study, the EM method is utilized to assign weights to evaluation indicators based on their intrinsic variability. By quantifying the influence of each indicator objectively, this method removes subjectivity, ensuring that indicators with higher variability are assigned greater weights (32).

The Coupling Coordination Degree Model (CCDM) has become a central tool for assessing synergistic interactions among systems such as economic development, environmental governance, and social progress (33). CCDM quantifies the degree of coordination between systems, highlights imbalances, and informs targeted policy interventions (34). This model will be applied to calculate the coupling and coordination degree at the regional level, specifically addressing the challenges faced by mountainous and island areas in comparison to urban centers. By providing a detailed understanding of system dynamics and efficiency, this approach aligns with the study’s goal of exploring system interactions in Zhejiang Province’s mountainous and island areas compared to other regions.

The fsQCA model identifies causal pathways leading to high or low coupling and coordination by analyzing configurations of multiple conditions. In healthcare research, fsQCA has been used to examine the efficiency of medical institutions (35) used fsQCA to identify configurations of factors influencing technical efficiency in China’s medical institutions, revealing multiple pathways to achieving high efficiency. While the traditional static fsQCA method is effective in analyzing concurrent relationships among multiple factors, it does not account for the dynamic evolution of configuration outcomes. Since the impact of policy implementation is gradual, unfolding over time (36), it is necessary to incorporate the time factor into the QCA to explore the dynamic evolution of configurations. Recent studies have introduced the time factor into QCA. Garcia summarized and calibrated multi-stage sample data (37), while Song et al. (38) examined how technological, organizational, and environmental factors drive enterprise digital innovation under dynamic fsQCA. Similarly, Jiang et al. (39) explored the determinants of low-carbon logistics capability using dynamic fsQCA.

In contrast, the multi-stage qualitative comparative analysis method uncovers the evolutionary trends of conditions or configurations over time, revealing how configurations change across multiple stages. If a configuration remains stable over several periods, it suggests that certain paths have played a dominant role during those times. To further validate the QCA findings with longitudinal qualitative evidence, this study integrates data from 25 mountainous and island regions over a three-year period (2020–2022). During this period, regional healthcare equity evolved with the implementation of *Mountain-Sea Cooperation Project*. The systematic approach of dynamic fsQCA makes it a powerful tool for analyzing multifaceted phenomena. This paper uses multi-stage dynamic fsQCA to analyze the collective relationships and joint effects of economic, social, and healthcare factors, identifying pathways to improve regional healthcare equity and exploring the evolution of these paths over time. This combined methodological approach enhances the robustness of the results by offering both configurational insights and a temporal contextualization of policy impacts. To more effectively reveal pathways to achieving regional healthcare equity in mountainous and island areas, the study designates the period from 2020 to 2022 as the research time window. This period is divided into three distinct time frames: 2020, 2021, and 2022, representing the stages before, during, and after the implementation of the policy.

This study employs a combination of the CCDM and fsQCA to assess the coupling coordination and to identify causal pathways within the interactions among economic, social, and healthcare systems in Zhejiang Province. Existing applications of the CCDM are often integrated with regression-based methods and machine learning algorithms derived from such frameworks (40). In parallel, fsQCA has been increasingly combined with Structural Equation Modeling (SEM) approaches (41). These methodologies offer the advantage of identifying influencing variables and quantifying their respective impacts. However, they typically involve complex computational procedures and require large sample sizes to ensure the stability and reliability of results. Moreover, SEM-based techniques necessitate the prior specification of potential influencing factors, which may not align with exploratory research contexts. Given the relatively small sample size in this study and the need for precise exploration of interaction pathways and conditional relationships, these conventional methods are less suited to the analytical objectives of the present research.

Nevertheless, the combination of fsQCA and CCDM offers distinct methodological advantages. fsQCA is particularly effective in handling smaller sample sizes, a domain where machine learning approaches generally require extensive datasets. Furthermore, fsQCA can accommodate mixed data types (both qualitative and quantitative), which is crucial in practical applications where heterogeneous data sources are common. Unlike machine learning models, which often require additional interpretive tools such as SHAP (Shapley Additive Explanations) values to explain model outputs, fsQCA directly provides clear and actionable condition-combination formulas. These formulas facilitate the straightforward mapping of policy interventions without the necessity for secondary modeling or extensive post-hoc explanation. This inherent simplicity renders fsQCA particularly advantageous for policy design, especially when research findings need to be translated into specific, implementable interventions.

### 3.4 Research methods

#### 3.4.1 Entropy method

**Step 1:** The date goes through dimensionless processing. To eliminate the incommensurability of the indicators typically have different units and scales. This study use the extreme-value methods to scale the data into a dimensionless range (e.g., [0, 1]) while retaining proportional relationships among the values. The data standardization formula is Li et al. (42):


(1) $$\text{For positive indicators}:x_{\mathrm{ijt}}=\frac{x_{\mathrm{ijt}}-x_{\text{ijtmin}}}{x_{\text{ijtmax}}-x_{\mathrm{ijt}}}$$

(2) $$\text{For negative indicators}:x_{\mathrm{ijt}}=\frac{x_{\text{ijtmax}}-x_{\mathrm{ijt}}}{x_{\text{ijtmax}}-x_{\text{ijtmin}}}$$

where $x_{\mathrm{ijt}}$ represents the original values of indicator j in alternative i and year t, $x_{\text{ijtmax}}=\max\{x_{1\mathrm{jt}},x_{2\mathrm{jt}},x_{\mathrm{mjt}}\}$ and $x_{\text{ijtmin}}=\min\{x_{1\mathrm{jt}},x_{2\mathrm{jt}},x_{\mathrm{mjt}}\}$ , and $x_{\mathrm{ijt}}\in [0,1]$ .**Step 2:** To ensure all indicators are non-negative before applying the EM, as the calculation of proportions and entropy values requires positive or zero values. This step is critical for avoiding mathematical errors and ensuring accurate weight calculation (43). Calculate the characteristic proportion of the indictor *j* in the alternative *i* and year *t* (*m* presents the total number of alternatives in each year):


(3) $$p_{\mathrm{ijt}}=x_{\mathrm{ijt}}/\sum_{i=1}^{m}x_{\mathrm{ijt}}$$

**Step 3:** Calculate the entropy value of indicator *j* in year *t* (44):


(4) $$E_{\mathrm{jt}}=-\frac{1}{\ln m}\sum_{i=1}^{m}p_{\mathrm{ijt}}\ln(p_{\mathrm{ijt}})$$

**Step 4:** Calculate the weight of indicator j in year t, the $w_{\mathrm{jt}}$ represent the comprehensive evaluation index of each subsystem (45):


(5) $$w_{\mathrm{jt}}=\frac{1-E_{\mathrm{jt}}}{\sum_{j=1}^{n}(1-E_{\mathrm{jt}})}$$

**Step 5:** Calculate the subsystem’s comprehensive quality score:


(6) $$\begin{array}{c} U_{\text{ECit}}=\sum_{j=1}^{7}w_{\mathrm{jt}}x_{\mathrm{ijt},}\\ U_{\text{SOit}}=\sum_{j=8}^{12}w_{\mathrm{jt}}x_{\mathrm{ijt},}\\ U_{\text{HEit}}=\sum_{j=13}^{20}w_{\mathrm{jt}}x_{\mathrm{ijt},} \end{array}$$

#### 3.4.2 Coupling coordination model

The coupling degree refers to the interaction and influence between two or more systems, reflecting the degree of interdependence and constraints between systems. This study uses a coupled coordination model to calculate the coupled coordination scheduling between Economy, Society and Healthcare system (46).

**Step 1:** Calculate the coupling degree. The model is mathematically expressed as follows:


(7) $$C=3\times {\left\{\frac{U_{\mathrm{EC}}\times U_{\mathrm{SO}}\times U_{\mathrm{SO}}}{{\left[U_{\mathrm{EC}}+U_{\mathrm{SO}}+U_{\mathrm{HE}}\right]}^{3}}\right\}}^{\frac{1}{3}}$$

**Step 2:** Calculate the coupling coordination degree. The coupling coordination degree is calculated as below:


(8) $$D=\sqrt{C\times T\;},T=\alpha U_{\mathrm{EC}}+\beta U_{\mathrm{SO}}+\gamma U_{\mathrm{SO}}$$

where the D represents the coupling coordination degree of the ESH system, The coupling degree D, ranging from 0 (Extreme disorder) to 1 (Excellent Coordination), and α , β , γ , denote subsystem’s weight coefficient, which are 1/3 in this study. According to the classification standard (47), the coupling coordination degree can be divided into ten grades, as shown in Table 2.

**Table 2:** Classification of the coupling coordination degree.

Coupling coordination degree	Grade	Contextualisation
[0.0 ~ 0.1]	Extreme disorder	The economic, social, and healthcare systems are severely disjointed, with no meaningful interaction or mutual reinforcement, reflecting profound systemic dysfunction often seen in regions facing extreme underdevelopment or major crises.
[0.1 ~ 0.2]	Severe disorder	Basic elements of subsystems may exist independently, but interactions are minimal, and developments in one domain often exacerbate weaknesses in others, leading to persistent instability.
[0.2 ~ 0.3]	Moderate disorder	Certain subsystems show preliminary development; however, contradictions between them are significant, hindering the formation of stable synergistic growth and resulting in fragmented service delivery.
[0.3 ~ 0.4]	Mild disorder	Subsystems begin to exhibit limited coordination, yet connections remain weak and uneven, requiring initial but substantial efforts toward integrated planning and policy support.
[0.4 ~ 0.5]	Near disorder	Some degree of interaction is emerging among the subsystems, but substantial structural imbalances and mismatches still prevent the realization of effective coordination.
[0.5 ~ 0.6]	Reluctant coordination	Basic systemic cooperation has started, but the synergy is unstable and easily disrupted, requiring focused interventions and cross-sector collaboration to consolidate progress.
[0.6 ~ 0.7]	Primary coordination	The economic, social, and healthcare systems are forming structured, stable links, although integration depth remains limited and continued optimization efforts are necessary.
[0.7 ~ 0.8]	Intermediate coordination	Subsystems interact cohesively with visible mutual promotion effects, creating a relatively stable and synergistic development pattern across domains.
[0.8 ~ 0.9]	Good coordination	Strong systemic integration is achieved, with economy, society, and healthcare sectors reinforcing each other, enhancing both the quality and efficiency of regional development.
[0.9 ~ 1.0]	Excellent coordination	Full, adaptive integration is realized, with dynamic feedback mechanisms among subsystems driving self-sustaining, high-quality development, representing an ideal model for regional synergy.

#### 3.4.3 fsQCA analysis

The configuration of the ESH system affecting healthcare equity is a complex process influenced by multiple factors. Since these factors are interdependent, the QCA method is well-suited to handle such complex causal relationships. fsQCA analysis involves several key steps to uncover these intricate causal linkages. Based on the weight coefficient ordering derived from the entropy weight method and considering the limited number of cases, we excluded the first-order variables Z2, Z3, and Z4. This step ensures the robustness and accuracy of the analysis by focusing on the most relevant variables.

**Step 1:** We averaged the normalized data within each subsystem to represent the value of each subsystem. Due to the polarization of regional development in Zhejiang Province, raw data are calibrated into fuzzy set membership scores using quartiles (75th, 50th, 25th) (48, 49), representing full membership, the crossover point, and full non-membership, respectively (49).

**Step 2:** The analysis is to identify attributes that are required for the outcome of interest to take on a high value. This is termed as a necessary condition in fsQCA analysis, A condition is considered “necessary” if its associated consistency and coverage are > = 0.9 and > = 0.5, respectively (50). Next, necessary conditions are identified by calculating consistency and coverage metrics. Consistency values above 0.9 indicate a strong relationship, while coverage measures the scope of cases explained by the condition (51).

**Step 3:** Given the inherent asymmetry of fsQCA, the analysis was conducted separately for both the presence and absence of the outcome. To assess sufficiency, a frequency benchmark of ≥1 was applied, along with a raw consistency threshold of ≥0.80. Additionally, the proportional reduction in inconsistency (PRI) cutoff was determined using a natural break approach (52).

**Step 4:** In fsQCA, three possible sets of solutions (complex, parsimonious, and intermediate solutions) can be calculated based on how we interpret the configurations missing in the data and our assumptions based on the available general knowledge Parsimonious solutions include all logical remainders, often leading to overly simplified results. Intermediate solutions consider only those logical remainders that align with theoretical expectations, producing relatively simplified outcomes. In contrast, complex solutions exclude all logical remainders, resulting in more detailed and intricate outcomes. This study references both intermediate and parsimonious solutions for its analysis. Logical minimization simplifies the truth table and identifies parsimonious solutions that explain the outcome. Finally, a robustness test is conducted to improve the reliability of the results.

## 4 Result

### 4.1 Coupling coordination degree of the ESH system

#### 4.1.1 The region characteristic of coupling coordination degree

**Figure 2:**
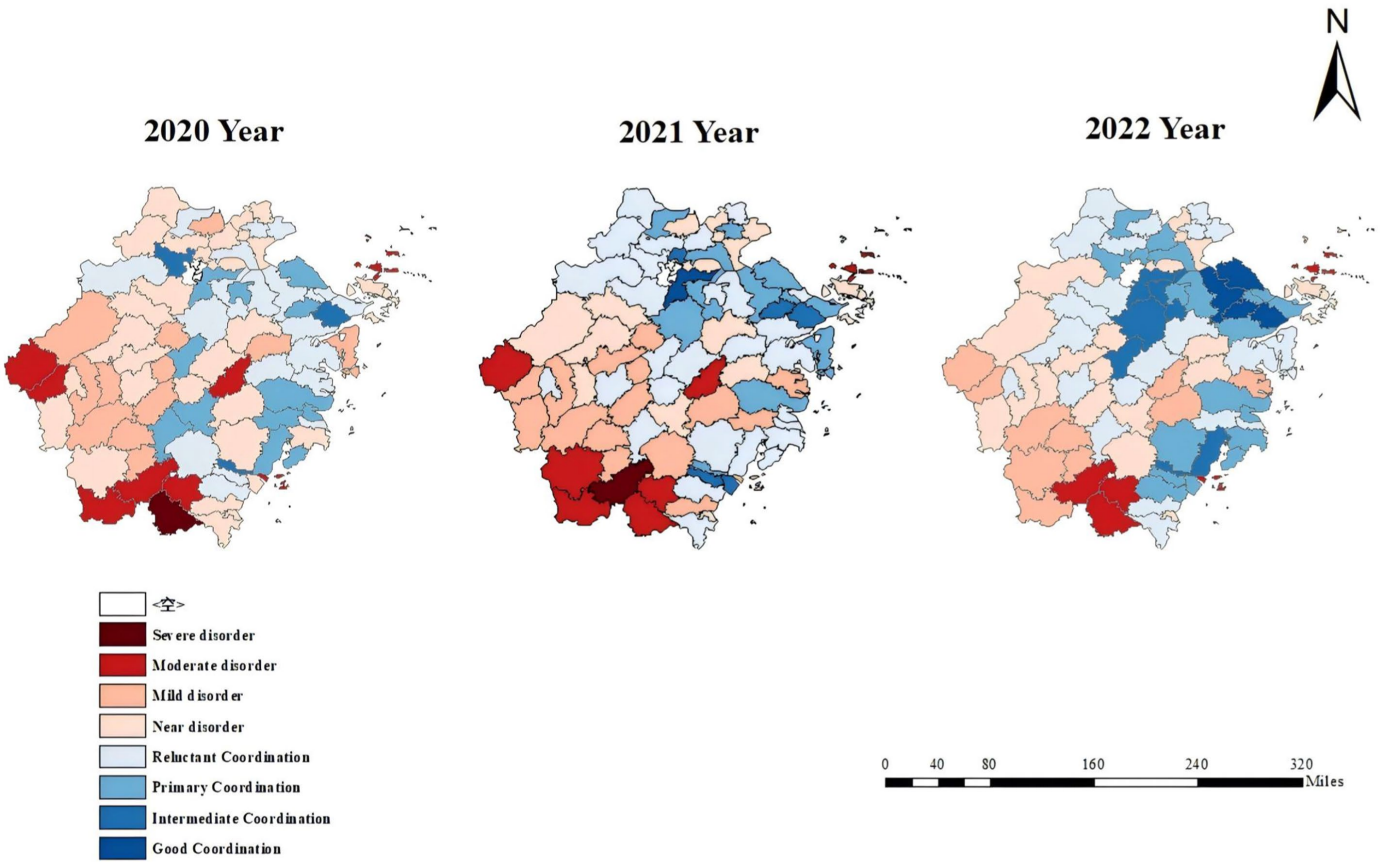
The evolution plots of the CCD across 2020, 2021 and 2022 years.

Region analysis identifies key intervention areas, highlights the importance of regional linkages in driving development, and supports the optimization of spatial planning to guide the formulation of differentiated regional policies. In 2022, the average coupling coordination degree (CCD) across 86 counties in Zhejiang Province was 0.536, with a median value of 0.557. Approximately 35% of counties had a coordination degree below the average, while 48% were classified between Reluctant Coordination and Primary Coordination, reflecting an an overall level of *Reluctant Coordination*, with an internal coefficient of variation (CV) of 0.276. Table 3 indicates that, on average, the CCD in the *Mountain/Island regions* (0.382) was significantly lower than in other regions (0.599), remaining in the *Mild Disorder*. Regions such as *Dongtou*, *Wencheng*, *Taishun*, and *Daishan* remained at a relatively low level of *Moderate Disorder*, suggesting a developmental gap between the *Mountain/Island* areas and other regions. The CV for the *Mountain/Island* regions (0.27) was notably higher than that of other regions (0.19), indicating greater internal development disparities. As observed in the topographic map in Figure 2, the four regions in *Moderate Disorder* are located in mountainous and island areas. In contrast, *Yinzhou*, *Haishu*, *Cixi*, and *Yuyao*, located in more economically developed areas, achieved high levels of *Good Coordination.*

**Table 3:** Regional comparison of statistical indicators across years 2020–2022.

Year	Mean		CV		Std		Median	
	Mountain/Island	Other_Regions	Mountain/Island	Other_Regions	Mountain/Island	Other_Regions	Mountain/Island	Other_Regions
2020	0.355	0.504	0.118	0.104	0.331	0.206	0.318	0.504
2021	0.363	0.554	0.097	0.107	0.268	0.192	0.370	0.545
2022	0.382	0.599	0.103	0.114	0.270	0.190	0.380	0.590

#### 4.1.2 The temporal trend of the coupling coordination degree

Analyzing temporal trends is crucial for evaluating dynamic changes in policy implementation and regional imbalances, forming the foundation for policy evaluation and improvement. Equations 6–8 were employed to calculate the Coupling Coordination Degree (CCD) of the economic, social, and healthcare (ESH) systems across various cities in Zhejiang Province. From 2020 (0.468) to 2022 (0.536), the average CCD steadily increased, with internal variation stabilizing over the 3 years in whole areas. The number of regions reaching Intermediate Coordination or above increased 2.3-fold, while those in Mild Disorder or below decreased by 27%. Temporal changes in CCD values for mountainous and island regions from 2020 to 2022 are presented in Table 3, indicate gradual improvement, although these regions consistently lagged behind other areas, highlighting persistent developmental disparities. The average CCD in the mountainous/island regions shifted from *Mild Disorder* (0.355) to *Near Disorder* (0.382), showing positive development. As shown in Figure 2, the CCD exhibits a distinct low in the southwest, high in the northeast distribution and evolutionary pattern. In 2020, several southwestern mountainous areas (e.g., *Wencheng, Qingyuan, Jingning*) and northeastern island regions (e.g., *Shengsi, Daishan*) were in a state of *Moderate Disorder*. Conversely, economically developed areas such as *Yuhang*, *Yinzhou*, and *Lucheng* achieved *Primary Coordination.* In 2021, there was a significant improvement in the CCD across all counties, with the range of areas classified as *Reluctant Coordination* expanding, with *Xiaoshan* (a developed areas) advancing to *Good Coordination*. Meanwhile, the number of regions in *Moderate Disorde*r (e.g., *Shengsi, Dongtou*) decreased, particularly in *mountainous/island areas*. By 2022, CCD levels across counties had markedly advanced (Table 4). The number of regions achieving *Intermediate Coordination* and *Good Coordination* increased significantly. The three major cities—*Hangzhou*, *Ningbo*, and *Wenzhou*—maintained consistently high coordination levels and played a significant role in driving the development of adjacent cities. Relying on these metropolitan clusters, surrounding regions experienced substantial improvements in coordination, forming a continuous high-value spatial pattern. However, regions in *Near Disorder* remained concentrated in economically underdeveloped cities such as *Lishui* and *Quzhou*. Some mountainous and island regions, like *Daishan* and *Wencheng*, persisted in a state of *Moderate Disorder*.

**Table 4:** Temporal changes in coupling coordination.

City	2020_CCD	2021_CCD	2022_CCD
Xiaoshan	0.647	0.85	0.794
Yuhang	0.739	0.569	0.673
Linping	0.448	0.705	0.605
Qiantang	0.448	0.471	0.453
Fuyang	0.485	0.599	0.557
Linan	0.521	0.52	0.487
Tonglu	0.404	0.498	0.525
Chunan	0.323	0.413	0.439
Jiande	0.406	0.496	0.516
Haishu	0.654	0.745	0.807
Jiangbei	0.524	0.634	0.621
Beilun	0.539	0.655	0.613
Zhenhai	0.541	0.592	0.696
Yinzhou	0.764	0.72	0.864
Fenghua	0.535	0.565	0.62
Xiangshan	0.377	0.64	0.567
Ninghai	0.532	0.546	0.571
Yuyao	0.548	0.695	0.8
Cixi	0.631	0.626	0.802
Lucheng	0.737	0.651	0.773
Longwan	0.472	0.711	0.635
Ouhai	0.552	0.741	0.644
Dongtou	0.259	0.34	0.24
Yongjia	0.442	0.572	0.657
Pingyang	0.487	0.378	0.571
Cangnan	0.406	0.547	0.553
Wencheng	0.233	0.239	0.221
Taishun	0.197	0.276	0.224
Ruian	0.572	0.534	0.69
Yueqing	0.603	0.53	0.736
Longgang	0.417	0.448	0.413
Nanhu	0.587	0.62	0.555
Xiuzhou	0.419	0.487	0.498
Jiashan	0.494	0.506	0.585
Haiyan	0.445	0.465	0.478
Haining	0.561	0.649	0.68
Pinghu	0.525	0.455	0.569
Tongxiang	0.472	0.583	0.61
Wuxing	0.591	0.645	0.671
Nanxun	0.386	0.5	0.57
Deqing	0.439	0.551	0.561
Changxing	0.465	0.521	0.59
Anji	0.417	0.527	0.556
Yuecheng	0.616	0.566	0.644
Keqiao	0.599	0.615	0.731
Shangyu	0.504	0.587	0.641
Xinchang	0.365	0.53	0.455
Zhuji	0.534	0.696	0.741
Shengzhou	0.474	0.44	0.569
Wucheng	0.463	0.588	0.594
Jindong	0.374	0.378	0.471
Wuyi	0.318	0.386	0.421
Pujiang	0.38	0.4	0.438
Panan	0.278	0.286	0.38
Lanxi	0.417	0.373	0.478
Yiwu	0.631	0.523	0.74
Dongyang	0.495	0.525	0.559
Yongkang	0.454	0.543	0.532
Kecheng	0.436	0.545	0.577
Qujiang	0.339	0.37	0.455
Changshan	0.264	0.361	0.393
Kaihua	0.266	0.245	0.326
Longyou	0.312	0.442	0.493
Jiangshan	0.416	0.364	0.456
Dinghai	0.446	0.452	0.491
Putuo	0.431	0.444	0.426
Daishan	0.212	0.262	0.245
Shengsi	0.257	0.439	0.311
Jiaojiang	0.609	0.512	0.603
Huangyan	0.617	0.382	0.52
Luqiao	0.558	0.517	0.539
Sanmen	0.537	0.347	0.321
Tiantai	0.537	0.483	0.438
Xianju	0.479	0.382	0.321
Wenling	0.458	0.581	0.69
Linhai	0.679	0.603	0.651
Yuhuan	0.617	0.585	0.641
Liandu	0.622	0.526	0.6
Qingtian	0.507	0.387	0.441
Jinyun	0.629	0.434	0.444
Suichang	0.301	0.39	0.379
Songyang	0.3	0.373	0.393
Yunhe	0.341	0.333	0.325
Qingyuan	0.233	0.224	0.31
Jingning	0.226	0.158	0.246
Longquan	0.486	0.276	0.372

### 4.2 Analysis of the driving factors of the ESH system’s coupling coordination degree

According to the results of the above data analysis, the development of mountain island region is the key to the overall coupling coordination degree. CCD improvements often result from the combined effects of multiple factors, By identifying the conditions leading to high and low CCD, fsQCA provides actionable insights for designing targeted interventions. The findings from the analysis of necessity are presented in Table 5. We test for necessary conditions both for the high CCD and for its negation (i.e., not high CCD or low/medium CCD) of the mountain/island areas. Table 6 presents a fuzzy-set qualitative comparative analysis (fsQCA) of configurations leading to high CCD and ~high CCD in Mountainous/Island Areas. The table identifies distinct configurations based on core and peripheral conditions across various economic and healthcare-related variables. In the following, we describe the inductively identified types of configurations leading to scoring high in CCD: *Social Security-Social Assistance Dual Driver Type (2020), Healthcare Service Accessibility-Healthcare Service Fairness Dual Driver Type (2021), Social Assistance-Healthcare Service Accessibility Dual Driver Type (2022), Social Assistance Constraint Type (2020), High Healthcare Service Accessibility Constraint Type (2020), Healthcare Service Accessibility Constraint Type (2021, 2022), Healthcare Service Fairness Constraint Type (2021).*

**Table 5:** Result of univariate necessity analysis of mountainous/island.

Subsystem	2020 year				2021 year				2022 year			
	High CCD		~High CCD		High CCD		~High CCD		High CCD		~High CCD	
	Consistency	Coverage	Consistency	Coverage	Consistency	Coverage	Consistency	Coverage	Consistency	Coverage	Consistency	Coverage
Economic aggregate (Z1)	0.609	0.270	0.412	0.866	0.310	0.800	0.044	0.800	0.609	0.270	0.412	0.866
~ Economic aggregate (Z1)	0.699	0.200	0.653	0.888	0.923	0.120	**0.989**	**0.910**	0.699	0.200	0.653	0.888
Social security (Z5)	0.775	0.227	0.587	0.815	0.168	0.598	0.040	1.000	0.775	0.227	0.587	0.815
~ Social security (Z5)	0.368	0.158	0.443	0.903	1.000	0.128	**0.984**	**0.893**	0.368	0.158	0.443	0.903
Social assistance (Z6)	0.678	0.327	0.312	0.715	0.787	0.200	0.500	0.897	0.678	0.327	0.312	0.715
~ Social assistance (Z6)	0.409	0.111	0.706	0.912	0.594	0.144	0.554	0.948	0.409	0.111	0.706	0.912
Healthcare service accessibility (Z7)	0.485	0.199	0.470	0.917	0.661	0.472	0.145	0.733	0.485	0.199	0.470	0.917
~ Healthcare service accessibility (Z7)	0.798	0.241	0.589	0.845	0.626	0.094	**0.895**	**0.949**	0.798	0.241	0.589	0.845
Healthcare service fairness (Z8)	0.828	0.298	0.471	0.803	0.939	0.321	0.332	0.800	0.828	0.298	0.471	0.803
~ Healthcare service fairness (Z8)	0.453	0.153	0.588	0.942	0.416	0.081	0.719	0.988	0.453	0.153	0.588	0.942

Bold font indicates that this condition is a necessary one.

**Table 6:** Analysis of sufficient conditions at consistency >0.80 and frequency = 1 (intermediate solution).

Configuration	2020 year						2021 year			2022 year		
	High CCD	~High CCD					High CCD	~High CCD		High CCD	~High CCD	
	1	1a	2a	3a	4a	5a	1	1b	2b	1	1c	2c
Economic aggregate (Z1)	⊗	⊗		•		⊗	⊗	⊗	⊗	⊗	⊗	⊗
Social security (Z5)	●		•	•	•	⊗	⊗	⊗	⊗	⊗	⊗	⊗
Social assistance (Z6)	●	**⊗**	**⊗**			**⊗**	•		•	●		**⊗**
Healthcare service accessibility (Z7)	**⊗**			●	●	⊗	●	**⊗**		●	**⊗**	
Healthcare service fairness (Z8)	•	⊗	⊗		•		●		**⊗**	•		•
Consistency	0.828	0.998	0.907	0.891	0.875	1.000	0.701	0.952	1.000	0.735	0.946	0.998
Raw coverage	0.255	0.415	0.275	0.334	0.297	0.314	0.545	0.889	0.344	0.519	0.908	0.393
Unique coverage	0.255	0.017	0.043	0.043	0.032	0.044	0.545	0.584	0.039	0.519	0.563	0.048
Solution consistency	0.828	0.918					0.701	0.954		0.735	0.948	
Solution coverage	0.255	0.795					0.545	0.928		0.519	0.956	

Black circles (•) indicate the presence of a condition, and circles with “x” (⊗) indicate its negation. Large circles indicate core conditions, and small ones represent peripheral.

#### 4.2.1 Testing for predictive validity

We conduct four different robustness checks. First we alter the consistency threshold to 0.75 (first robustness check) to conduct a sensitivity analysis, but only minor changes were observed. Second we increase the consistency threshold to 0.85 (second robustness check). Table 7 provides a summary of all *post hoc* robustness checks and the fit with our initial analysis (details available from authors). In the next step, we discuss and interpret these outputs to develop a better understanding of the resulting solution terms beyond the rather mechanical fsQCA software minimization process.

**Table 7:** Robustness check summary.

Solution	2020 year						2021 year			2022 year		
	High CCD	~High CCD					High CCD	~High CCD		High CCD	~High CCD	
	1	1a	2a	3a	4a	5a	1	1b	2b	1	1c	2c
Consistency ≧ 0.75	✓	✓	✓	✓	✓	✓	✓	✓	✓	✓	✓	✓
Consistency ≧ 0.80	✓	✓	✓	✓	✓	✓	✓	✓	✓	✓	✓	✓
Consistency ≧ 0.85	✓	✓	✓	✓	✓	✓	✓	✓	✓	✓	✓	✓

✓ is placed in cases where the same solution paths occurs. (✓) is placed where the solution term is comparable, but 1 condition is added or removed vs. initial analysis.

#### 4.2.2 Findings for high CCD configurations

##### 4.2.2.1 Social security-social assistance dual driver type (path 1 in 2020)

The first configuration is characterized by a combination of high social security and high social assistance. In this path, areas with both high social security and high social assistance can achieve a balanced allocation of healthcare resources, even in the absence of direct accessibility to healthcare services. This configuration accounts for approximately 25.5% of the cases, with 25.5% of mountainous and island regions reaching high CCD through this dual-driver model. A notable example of this path is *Tiantai*.

##### 4.2.2.2 Healthcare service accessibility-healthcare service fairness dual driver type (path 1 in 2021)

The second configuration is marked by a combination of high healthcare service accessibility and fairness. In *Path 1*, regions that exhibit both high accessibility to healthcare services and a fair distribution of these services can achieve equitable healthcare resource allocation, even in the absence of social assistance. This configuration explains about 54.5% of the cases, with 54.5% of the mountainous and island regions achieving high CCD through this pathway. Representative regions include *Cangnan.*

##### 4.2.2.3 Social assistance-healthcare service accessibility dual driver type (path 1 in 2022)

The third configuration is defined by a combination of high healthcare service accessibility and robust social assistance. In this path, regions that benefit from strong social assistance and high healthcare service fairness are able to achieve equitable allocation of healthcare resources, even when healthcare service fairness is relatively low. This configuration accounts for approximately 51.9% of cases, demonstrating that 51.9% of mountainous and island areas attain high CCD through this approach. A representative example of this configuration is *Cangnan*.

#### 4.2.3 Findings of asymmetric approaches

##### 4.2.3.1 Social assistance constraint type (path 1a 2a and 5a in 2020)

The fourth configuration highlights that the absence of high social assistance is a critical constraint to achieving high CCD. *Path 1a*, *2a and 5a* illustrate that without sufficient social assistance and with low social security, attaining high CCD becomes difficult. This constraint affects approximately 10.4% of cases, indicating that nearly one-tenth of mountainous and island areas face limitations due to poor healthcare fairness. Representative regions include *Taishun*.

##### 4.2.3.2 High healthcare service accessibility constraint type (paths 3a and 4a in 2020)

The fifth configuration highlights that excessively high healthcare service accessibility can limit high CCD. *Path 3a, 4a* demonstrate that very high levels of healthcare service accessibility can hinder the achievement of high CCD, even in the absence of other key conditions. Representative regions of this configuration is *Chunan.*

##### 4.2.3.3 Healthcare service accessibility constraint type (paths 1b in 2021)

The sixth configuration emphasizes the pivotal role of healthcare service accessibility in fostering high CCD. In this pathway, inadequate healthcare service accessibility emerges as a primary limitation, alongside insufficient social security, economic aggregate. This configuration explains 58.4% of cases, indicating that 58.4% of mountainous and island areas struggle with healthcare service accessibility. Representative regions include *Wencheng*.

##### 4.2.3.4 Healthcare service fairness constraint type (path 2b in 2021)

The seventh configuration identifies healthcare service fairness constraints preventing high CCD. Additional deficiencies in economic structure and social security further exacerbate these limitations. This pathway accounts for 3.9% of cases, suggesting that approximately 3.9% of mountainous and island areas are constrained by these factors. A representative region is *Taishun*.

##### 4.2.3.5 Healthcare service accessibility constraint type (paths 1c in 2022)

The eighth configuration underscores that low levels of healthcare service accessibility are key factors limiting CCD. *Path 1c* illustrates that inadequate healthcare service accessibility emerges as a primary limitation, the absence of sufficient social security and economic structure remains a barrier. This configuration explains 56.3% of cases, indicating that 56.3% of mountainous and island areas are constrained by these deficiencies. Representative regions include *Wencheng*.

##### 4.2.3.6 Social assistance constraint type (paths 2c in 2022)

*Paths 2c* illustrate that, despite the presence of healthcare service fairness, policy objectives cannot be fully realized without sufficient social assistance. These paths emphasize that the lack of social assistance acts as a critical barrier to achieving a high Coupling Coordination Degree (CCD). Even when initial progress is made in improving healthcare service fairness, the absence of adequate social assistance significantly undermines the pathway’s ability to drive high CCD outcomes. Representative regions include *Pan’an.*

## 5 Discussion

### 5.1 Theoretical contributions

This study provides significant theoretical contributions to the understanding of the coordinated development of economic, social, and healthcare systems. A key contribution is the integration of public service equalization into the established framework of coupling coordination, which has traditionally focused on the relationship between economic and healthcare systems (53). In contrast to conventional two-dimensional analytical models that focus solely on the coordination between economic development and healthcare equity (54), this research adopts a three-dimensional analytical perspective that simultaneously incorporates economic, social, and healthcare systems, By including the social dimension—specifically indicators reflecting social assistance and healthcare service accessibility—this study offers a more comprehensive understanding of the drivers and constraints affecting health equity in regional development. This approach contributes to the existing literature by addressing the previously underexplored role of social development in the coordinated advancement of health systems. Moreover, this study extends the application of coupling coordination theory to the regional level, with Zhejiang Province serving as a case study. This shift from macro-level national analyses to a localized context—such as the Mountain-Island Cooperation initiative—enables a deeper understanding of how regional policies and local interventions influence the interactions among economic, social, and healthcare systems. Additionally, the temporal analysis applied in this study demonstrates that the dynamics of system coordination evolve over time, rather than following a linear progression. This finding underscores the importance of continuous policy adjustments and local interventions in shaping the trajectory of system development.

### 5.2 Practical significance

#### 5.2.1 Temporal trends and coordination improvements

Between 2020 and 2022, Zhejiang Province experienced a consistent rise in its coupling coordination degree (CCD), signaling improved alignment across its economic, social, and healthcare systems. This progress can be attributed to several key factors. For example, the expansion of telemedicine (55) and the establishment of regional medical consortia (56) facilitated resource sharing and enhanced healthcare access in remote areas. Additionally, increased fiscal investments in healthcare (57), alongside strengthened social welfare programs (58), contributed to the shift from imbalance to greater coordination in many regions. The implementation of targeted policies, such as the *Healthy Zhejiang 2030 initiative* and the *Medical Consortium* reform, played a crucial role in improving resource allocation efficiency and fostering the integration of healthcare services (59).

#### 5.2.2 Regional disparities and spillover effects

Although overall improvements in coupling and coordination have been observed, substantial regional disparities persist, particularly in mountainous and island areas. These regions continue to lag behind in coupling and coordination coefficients and exhibit greater internal variability, as evidenced by higher differentiation coefficients. This highlights the persistent challenges of achieving healthcare equity in geographically and economically disadvantaged areas. Key issues include uneven distribution of healthcare resources and geographical barriers that hinder infrastructure development and service delivery. Furthermore, limited economic capacity and lower population density compound these challenges, leading to slower progress relative to urban centers. In contrast, cities such as Hangzhou and Ningbo, which are characterized by advanced levels of economic and social development, exhibit significantly higher coupling and coordination scores. In the dataset for Hangzhou, data for the districts of *Xihu*, *Gongshu*, and *Binjiang* were excluded. This decision was based on the fact that these districts underwent administrative boundary adjustments during the study period, resulting in inconsistencies in statistical definitions and data comparability. At the same time, other districts within Hangzhou—namely *Yuhang*, *Xiaoshan*, and *Shangcheng*—possess comparable levels of economic and social development and are sufficiently representative of the overall development status of the city. The average values for Hangzhou after excluding Xihu, Gongshu, and Binjiang do not show substantial deviation from the overall municipal average. Therefore, it is reasonable to conclude that the exclusion of these districts is unlikely to have introduced bias or significantly affected the overall findings of this study.

These urban areas function as regional growth hubs, generating positive spillover effects that benefit adjacent counties and districts. Specifically, the diffusion of advanced medical technologies, modern management practices, and financial resources from core cities like Hangzhou and Ningbo has notably strengthened healthcare capabilities in neighboring regions. This spillover effect is facilitated by mechanisms such as inter-regional collaboration, infrastructure connectivity, and knowledge transfer (60). Consistent with prior studies (61, 62), our results confirm that significant internal disparities persist even in economically developed provinces such as Zhejiang. While earlier research emphasized the role of economic growth in promoting healthcare access, our findings suggest that economic development alone does not guarantee coordinated system advancement unless accompanied by improvements in social assistance and equitable resource distribution, This complements Chen, Zhenhua’ (63) argument that socio-economic infrastructure plays a mediating role in achieving health equity across regions.

#### 5.2.3 Analysis of driving factors based on fsQCA

The dynamic QCA analysis of multi-factor influences in the Mountain-Sea Improvement areas indicates that social assistance and healthcare accessibility consistently emerge as the most significant positive drivers of coordinated development, while lagging economic development remains the primary negative factor. In Zhejiang Province, which exhibits relatively advanced development, the region’s progress has evolved in stages, starting with improvements in healthcare security through social insurance systems, followed by enhanced healthcare service accessibility, and culminating in a focus on comprehensive health coverage, with a growing emphasis on inclusive economic development.

This finding supports the notion that economic vitality in developed regions facilitates continued investment in healthcare innovation and capacity building, which, in turn, benefits less-developed areas through collaborative networks (64). The emphasis on social assistance and public service equality underscores the region’s commitment to addressing the needs of vulnerable populations, including those suffering from chronic diseases and rare conditions (65). As such, the development of social security systems has become a critical positive factor in promoting coordination, improving the breadth and depth of UHC.

In 2021, the region’s health administration departments implemented targeted capacity-building programs for healthcare institutions in the Mountain-Sea Improvement areas, increasing personnel and resource investments. These efforts have further improved healthcare accessibility and fairness—critical components in assessing the width of UHC. As these initiatives continue, the development of primary healthcare human resources remains a crucial focus for further improving healthcare accessibility. In response, the region has introduced policies to strengthen personnel recruitment and capacity building (66). Moreover, as a provincial demonstration area for China’s common prosperity policy, the region continues to prioritize the protection of vulnerable groups, making healthcare accessibility and social assistance the central influencing factors in this context.

In regions with relatively low coordination, the primary constraints to development are economic conditions and inadequate social security systems, highlighting the need to improve regional development balance and address disparities between different population groups. In response, local governments have proposed a series of initiatives aimed at promoting common prosperity, including narrowing urban–rural gaps, regional inequalities, and income disparities (67, 68). At the same time, improvements in the fairness and accessibility of healthcare services have begun to demonstrate a positive impact on coordination, emerging as key drivers of sustainable development.

#### 5.2.4 Counterintuitive findings and implications

Notably, the fsQCA results reveal several counterintuitive configurations. One particularly important finding is that high healthcare accessibility, rather than strengthening the level of coupling coordination, may under certain conditions constrain it. This paradox indicates that simply increasing the number of healthcare facilities or medical personnel does not necessarily lead to better system coordination or improvements in health equity.

A likely explanation lies in the spatial and structural allocation of healthcare resources. Enhancing healthcare accessibility often depends on centralized fiscal investments and concentrated resource deployment. However, when such investment strategies fail to consider regional balance, they may inadvertently exacerbate urban–rural disparities or widen developmental gaps between regions. Without alignment to scientific allocation mechanisms, these investments may result in resource inefficiencies or the so-called “Matthew effect,” where advantaged areas continue to accumulate benefits while disadvantaged areas fall further behind. Evidence from international studies supports this interpretation. In Ethiopia, for instance, despite increased national investments in maternal health services, the difference in antenatal visit rates between affluent and impoverished households remained as large as 43 percentage points. In addition, nomadic communities in eastern Ethiopia faced persistent barriers to access due to geographical isolation and cultural differences. In the United States, the American Hospital Association has reported that despite rising healthcare expenditures, Medicare reimbursement rates cover only approximately 83 percent of the cost of care, creating significant financial pressures for healthcare institutions and indirectly limiting service accessibility, particularly in less affluent areas.

In regions where the healthcare supply is relatively abundant but economic and social foundations are weak, resource allocation tends to favor core urban centers while marginalizing peripheral communities. This pattern has been described in previous studies as an “inverted triangle” structure, where healthcare accessibility appears high at an aggregate level but fails to deliver meaningful access to vulnerable or remote populations. When improvements in healthcare infrastructure are not accompanied by equivalent progress in economic development (69) and social services, the marginal benefits of expanding healthcare inputs tend to decline. In some cases, disparities may not only persist but may actually deepen (70).

This highlights the crucial distinction between nominal accessibility, which refers to the presence of healthcare resources, and effective accessibility, which considers whether services are equitably distributed, financially affordable, and culturally appropriate for different population groups (71). Fragmented intersectoral policy implementation and over-concentration of healthcare infrastructure, when coupled with uneven regional development, may reduce the practical effectiveness of increased service supply. For example, primary healthcare institutions in some areas face the challenge of having healthcare personnel without permanent staffing structures, resulting in unstable service delivery. This form of superficial equalization in resource allocation can also displace essential investments in other public sectors, such as education and infrastructure, thereby hindering the region’s overall economic balance.

The fsQCA configuration findings further demonstrate that when economic and social systems fail to develop in a coordinated manner, misaligned development across sectors may exacerbate systemic inefficiencies and reinforce inequality. These insights underscore the importance of integrated and multi-sectoral policy strategies. Investments in healthcare infrastructure must be complemented by parallel improvements in economic empowerment, educational access, transportation networks, and social protection in order to generate synergistic effects and promote inclusive regional development.

### 5.3 Limitations and future research directions

Despite the valuable insights provided, this study acknowledges several limitations that offer opportunities for future research to further refine the understanding of regional development dynamics and healthcare equity.

#### 5.3.1 Geographic scope and data availability

This study’s geographic focus on Zhejiang Province, although representative of high-quality development in China, may not fully capture the diverse socio-economic and healthcare conditions found across other provinces, particularly in less developed areas. Future research should consider expanding the geographic scope to include additional provinces, particularly those in central and western China, to enhance the generalizability of the findings. Moreover, inconsistencies in the data due to administrative restructuring in certain districts highlight the need for future studies to address data gaps and apply the analytical framework to regions with more consistent and complete data sets.

#### 5.3.2 Temporal constraints and policy evolution

The three-year period of analysis (2020–2022) may not provide a comprehensive view of the long-term impacts of the Mountain-Island Cooperation project. Future research could benefit from extending the temporal scope to examine the long-term effects of regional healthcare policies and socio-economic changes. Such an extension would allow for a more nuanced understanding of how policy evolution unfolds over time, potentially uncovering delayed effects that would offer valuable insights into the dynamics of healthcare system integration with broader economic development.

#### 5.3.3 Limited dynamic analysis of configurations

While dynamic fuzzy-set qualitative comparative analysis (fsQCA) has provided valuable insights, future research could further expand this methodology by incorporating additional external factors, such as national economic policies, global health crises (e.g., COVID-19), and technological innovations. Integrating time-series analysis into the framework could enhance the understanding of the temporal dynamics between economic, social, and healthcare systems. This expanded approach would offer a deeper and more nuanced understanding of the factors driving the coupling coordination and its evolution over time, ultimately providing more robust and contextually relevant policy recommendations.

## 6 Conclusions and recommendations

### 6.1 Conclusion

The study highlights the significant disparities in coupling coordination between economic, social, and healthcare systems in Zhejiang Province, particularly between urban and mountainous/island regions. Over the period from 2020 to 2022, the overall coordination degree of these systems improved steadily, especially with increased public investments in healthcare, telemedicine, and social welfare programs. These efforts were pivotal in narrowing the gap between different regions, contributing to better healthcare service accessibility and equity. However, substantial regional disparities remain, with mountainous and island areas still lagging behind, underlining the need for targeted policy interventions.

The findings emphasize that high healthcare service accessibility, social assistance, and a fair distribution of healthcare services are critical factors driving the successful coordination of these systems. On the other hand, insufficient economic development and inadequate social security systems pose significant barriers to achieving high coordination, especially in less developed regions. The analysis also revealed that the coupling coordination degree (CCD) is not solely influenced by individual factors but is a result of the complex interplay of multiple conditions.

### 6.2 Recommendations

#### 6.2.1 Enhancing healthcare accessibility in disadvantaged regions

The findings underscore the urgent need to address persistent disparities in coupling coordination between urban centers and geographically disadvantaged regions, particularly mountainous and island areas. At the provincial level, policy efforts should move beyond merely increasing the number of healthcare facilities and instead prioritize improving effective healthcare accessibility. This entails ensuring that healthcare services are not only available but also equitably distributed, financially accessible, and responsive to local needs.

Targeted investments should focus on the establishment of satellite healthcare centers (72), the expansion of telemedicine networks (73), and the improvement of transportation infrastructure to overcome geographical constraints. Zhejiang Province’s Mountain-Island Cooperation Project provides an institutional foundation for implementing these strategies in a regionally tailored manner.

#### 6.2.2 Strengthening integrated social assistance systems

Beyond healthcare infrastructure, the results emphasize the critical role of social assistance programs in promoting health equity. Policy measures should aim to expand the coverage and increase the benefit levels of minimum living security programs, especially in low-income counties and districts (74). Moreover, integrating health insurance (75), basic pensions (76), and targeted social support into a comprehensive social protection system can mitigate socioeconomic vulnerabilities more effectively. Such integrated systems would align with international best practices and contribute to reducing systemic disparities in health outcomes by addressing broader social determinants of health.

#### 6.2.3 Leveraging international frameworks for holistic development

These insights support the integration of health equity goals into broader regional development strategies under the Common Prosperity Initiative. Establishing multi-sectoral coordination platforms—where economic, healthcare, and social policy sectors jointly plan and implement interventions—could enhance the alignment of economic growth with health equity objectives. International experiences offer valuable guidance: Norway’s Regional Development Program (77) focuses on balancing territorial disparities through coordinated economic and healthcare initiatives, while Canada’s telehealth expansion (78) and targeted federal transfers to remote regions demonstrate the effectiveness of integrated approaches. Drawing from such models, provinces like Zhejiang can embed healthcare equity more centrally into sustainable development frameworks, fostering more inclusive and resilient regional systems.

## Data Availability

Publicly available datasets were analyzed in this study. This data can be found at: https://tjj.zj.gov.cn/col/col1525563/index.html.
