# Supplementary material for: Coupling coordination and regional health equity: an empirical study of economic, social, and healthcare systems in Zhejiang Province, China
Source: Front Public Health. 2025 Jun 18;13:1581834. doi: 10.3389/fpubh.2025.1581834 (PMC12216669; doi:10.3389/fpubh.2025.1581834)
Supplement: Supplementary file 1 [file Supplementary_file_1.docx]

Fig.1.Study are of the Zhejiang in China


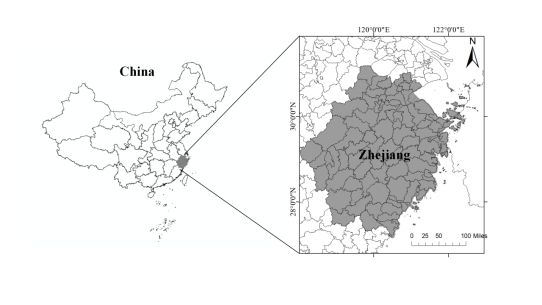


Table1:Specific indexes

| System | Subsystem | Indicator & Indicator type | Unit | Description | Reference |
| --- | --- | --- | --- | --- | --- |
| Economy | Economic Aggregate (Z1) | GDP [x1] | 100 Million Yuan | Gross Domestic Product |  |
|  |  | Fiscal Revenue [x2] | 100 Million Yuan | Local Government's Financial Status |  |
|  | Economic Structure (Z2) | Industrial Proportion in GDP [x3] | % | Distribution of Industrial Structure |  |
|  |  | Proportion of Tertiary Industry in GDP [x4] | % | Distribution of Industrial Structure |  |
|  | Economic Efficiency (Z3) | Per-capita GDP [x5] | Yuan | Comprehensive Economic Strength |  |
|  |  | Per-capita Disposable Income of Urban Residents [x6] | Yuan | Urban Resident Economic Conditions |  |
|  |  | Per-capita Disposable Income of Rural Residents [x7] | Yuan | Rural Resident Economic Conditions |  |
| Society | Public Investment (Z4) | General Public Service Expenditure [x8] | 100 Million Yuan | General Public Service |  |
|  |  | General Public Budget Expenditure [x9] | 100 Million Yuan | General Education Expenditure |  |
|  | Social Security (Z5) | Number of Urban and Rural Residents Participating in Basic Endowment Insurance[x10] | 10000 Persons | Basic Endowment Insurance Coverage |  |
|  |  | Persons Participating in the Unemployment Insurance Program [x11] | 10000 Persons | Unemployment Insurance Coverage |  |
|  | Social Assistance (Z6) | Number of Urban Residents in Minimum Living Security Program [x12] | Persons | Social Assistance |  |
| Healthcare | Accessibility (Z7) | Assistant Doctors per 1000 Residents [x13] | unit | Supply Capacity of Medical Services |  |
|  |  | Registered Nurses per 1000 Residents [x14] | unit | Supply Capacity of Medical Services |  |
|  |  | Number of Beds per 1000 Residents [x15] | unit | Supply Capacity of Medical Services |  |
|  |  | Urban and Rural Households Participating in the Basic Health Care Program [x16] | 10000 persons | Medical and Hygiene Guarantee |  |
|  |  | Birth Rate [x17] | ‰ | Birth Statistics |  |
|  | Fairness (Z8) | Number of Tertiary Hospitals [x18] | unit | Hospital Coverage |  |
|  |  | Number of Township Hospitals [x19] | unit | Community Health Services |  |
|  |  | Number of Village Clinics[x20] | unit | Community Health Services |  |

Table2: Classification of the coupling coordination degree

| Coupling Coordination Degree | Grade | Contextualisation |
| --- | --- | --- |
| [0.0 ~ 0.1) | Extreme Disorder | The economic, social, and healthcare systems are severely disjointed, with no meaningful interaction or mutual reinforcement, reflecting profound systemic dysfunction often seen in regions facing extreme underdevelopment or major crises. |
| [0.1 ~ 0.2) | Severe Disorder | Basic elements of subsystems may exist independently, but interactions are minimal, and developments in one domain often exacerbate weaknesses in others, leading to persistent instability. |
| [0.2 ~ 0.3) | Moderate Disorder | Certain subsystems show preliminary development; however, contradictions between them are significant, hindering the formation of stable synergistic growth and resulting in fragmented service delivery. |
| [0.3 ~ 0.4) | Mild Disorder | Subsystems begin to exhibit limited coordination, yet connections remain weak and uneven, requiring initial but substantial efforts toward integrated planning and policy support. |
| [0.4 ~ 0.5) | Near Disorder | Some degree of interaction is emerging among the subsystems, but substantial structural imbalances and mismatches still prevent the realization of effective coordination. |
| [0.5 ~ 0.6) | Reluctant Coordination | Basic systemic cooperation has started, but the synergy is unstable and easily disrupted, requiring focused interventions and cross-sector collaboration to consolidate progress. |
| [0.6 ~ 0.7) | Primary Coordination | The economic, social, and healthcare systems are forming structured, stable links, although integration depth remains limited and continued optimization efforts are necessary. |
| [0.7 ~ 0.8) | Intermediate Coordination | Subsystems interact cohesively with visible mutual promotion effects, creating a relatively stable and synergistic development pattern across domains. |
| [0.8 ~ 0.9) | Good Coordination | Strong systemic integration is achieved, with economy, society, and healthcare sectors reinforcing each other, enhancing both the quality and efficiency of regional development. |
| [0.9 ~ 1.0] | Excellent Coordination | Full, adaptive integration is realized, with dynamic feedback mechanisms among subsystems driving self-sustaining, high-quality development, representing an ideal model for regional synergy. |

Table3 Regional Comparison of Statistical Indicators Across Years 2020-2022

|  | mean | | CV | | std | | median | |
| --- | --- | --- | --- | --- | --- | --- | --- | --- |
| year | Mountain/Island | Other_Regions | Mountain/Island | Other_Regions | Mountain/Island | Other_Regions | Mountain/Island | Other_Regions |
| 2020 | 0.355 | 0.504 | 0.118 | 0.104 | 0.331 | 0.206 | 0.318 | 0.504 |
| 2021 | 0.363 | 0.554 | 0.097 | 0.107 | 0.268 | 0.192 | 0.370 | 0.545 |
| 2022 | 0.382 | 0.599 | 0.103 | 0.114 | 0.270 | 0.190 | 0.380 | 0.590 |


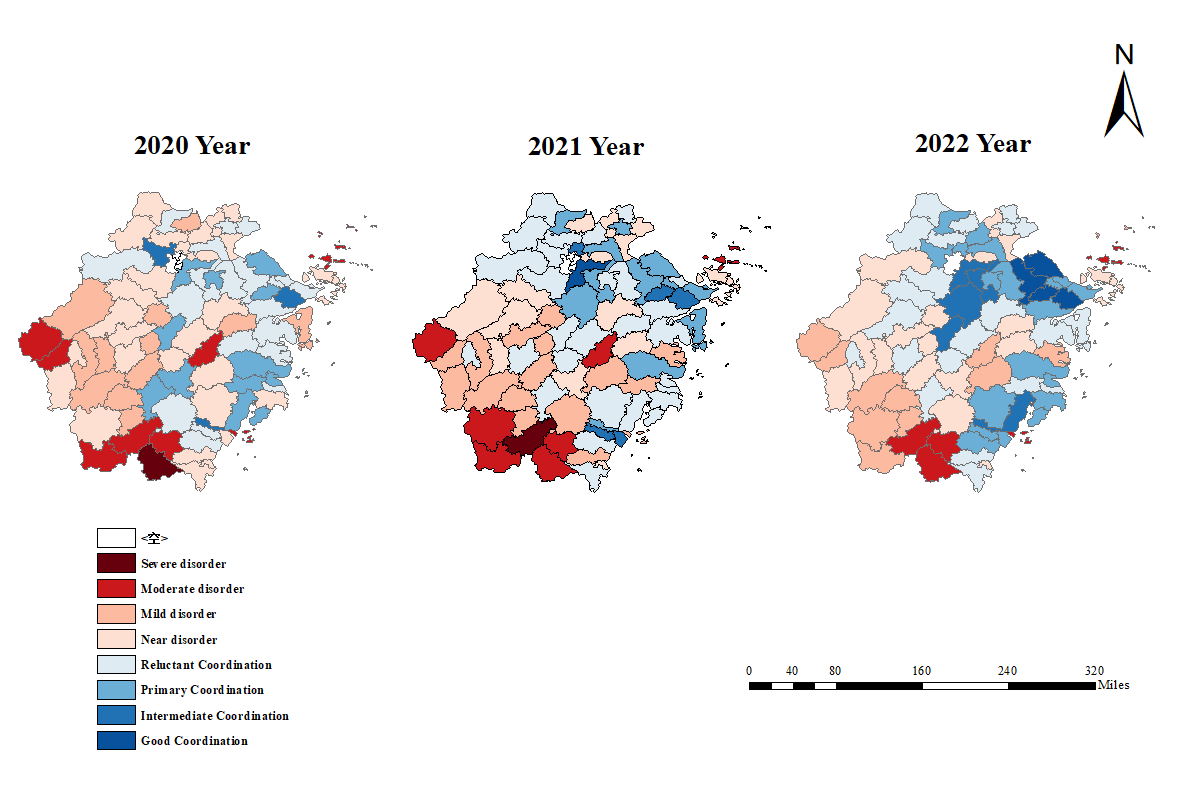


Fig.2.the evolution plots of the CCD across 2020,2021and 2022 years.

Table4:Temporal changes in couplinf coordination.

| city | 2020_CCD | 2021_CCD | 2022_CCD | city | 2020_CCD | 2021_CCD | 2022_CCD |
| --- | --- | --- | --- | --- | --- | --- | --- |
| Xiaoshan | 0.647 | 0.85 | 0.794 | Zhuji | 0.534 | 0.696 | 0.741 |
| Yuhang | 0.739 | 0.569 | 0.673 | Shengzhou | 0.474 | 0.44 | 0.569 |
| Linping | 0.448 | 0.705 | 0.605 | Wucheng | 0.463 | 0.588 | 0.594 |
| Qiantang | 0.448 | 0.471 | 0.453 | Jindong | 0.374 | 0.378 | 0.471 |
| Fuyang | 0.485 | 0.599 | 0.557 | Wuyi | 0.318 | 0.386 | 0.421 |
| Linan | 0.521 | 0.52 | 0.487 | Pujiang | 0.38 | 0.4 | 0.438 |
| Tonglu | 0.404 | 0.498 | 0.525 | Panan | 0.278 | 0.286 | 0.38 |
| Chunan | 0.323 | 0.413 | 0.439 | Lanxi | 0.417 | 0.373 | 0.478 |
| Jiande | 0.406 | 0.496 | 0.516 | Yiwu | 0.631 | 0.523 | 0.74 |
| Haishu | 0.654 | 0.745 | 0.807 | Dongyang | 0.495 | 0.525 | 0.559 |
| Jiangbei | 0.524 | 0.634 | 0.621 | Yongkang | 0.454 | 0.543 | 0.532 |
| Beilun | 0.539 | 0.655 | 0.613 | Kecheng | 0.436 | 0.545 | 0.577 |
| Zhenhai | 0.541 | 0.592 | 0.696 | Qujiang | 0.339 | 0.37 | 0.455 |
| Yinzhou | 0.764 | 0.72 | 0.864 | Changshan | 0.264 | 0.361 | 0.393 |
| Fenghua | 0.535 | 0.565 | 0.62 | Kaihua | 0.266 | 0.245 | 0.326 |
| Xiangshan | 0.377 | 0.64 | 0.567 | Longyou | 0.312 | 0.442 | 0.493 |
| Ninghai | 0.532 | 0.546 | 0.571 | Jiangshan | 0.416 | 0.364 | 0.456 |
| Yuyao | 0.548 | 0.695 | 0.8 | Dinghai | 0.446 | 0.452 | 0.491 |
| Cixi | 0.631 | 0.626 | 0.802 | Putuo | 0.431 | 0.444 | 0.426 |
| Lucheng | 0.737 | 0.651 | 0.773 | Daishan | 0.212 | 0.262 | 0.245 |
| Longwan | 0.472 | 0.711 | 0.635 | Shengsi | 0.257 | 0.439 | 0.311 |
| Ouhai | 0.552 | 0.741 | 0.644 | Jiaojiang | 0.609 | 0.512 | 0.603 |
| Dongtou | 0.259 | 0.34 | 0.24 | Huangyan | 0.617 | 0.382 | 0.52 |
| Yongjia | 0.442 | 0.572 | 0.657 | Luqiao | 0.558 | 0.517 | 0.539 |
| Pingyang | 0.487 | 0.378 | 0.571 | Sanmen | 0.537 | 0.347 | 0.321 |
| Cangnan | 0.406 | 0.547 | 0.553 | Tiantai | 0.537 | 0.483 | 0.438 |
| Wencheng | 0.233 | 0.239 | 0.221 | Xianju | 0.479 | 0.382 | 0.321 |
| Taishun | 0.197 | 0.276 | 0.224 | Wenling | 0.458 | 0.581 | 0.69 |
| Ruian | 0.572 | 0.534 | 0.69 | Linhai | 0.679 | 0.603 | 0.651 |
| Yueqing | 0.603 | 0.53 | 0.736 | Yuhuan | 0.617 | 0.585 | 0.641 |
| Longgang | 0.417 | 0.448 | 0.413 | Liandu | 0.622 | 0.526 | 0.6 |
| Nanhu | 0.587 | 0.62 | 0.555 | Qingtian | 0.507 | 0.387 | 0.441 |
| Xiuzhou | 0.419 | 0.487 | 0.498 | Jinyun | 0.629 | 0.434 | 0.444 |
| Jiashan | 0.494 | 0.506 | 0.585 | Suichang | 0.301 | 0.39 | 0.379 |
| Haiyan | 0.445 | 0.465 | 0.478 | Songyang | 0.3 | 0.373 | 0.393 |
| Haining | 0.561 | 0.649 | 0.68 | Yunhe | 0.341 | 0.333 | 0.325 |
| Pinghu | 0.525 | 0.455 | 0.569 | Qingyuan | 0.233 | 0.224 | 0.31 |
| Tongxiang | 0.472 | 0.583 | 0.61 | Jingning | 0.226 | 0.158 | 0.246 |
| Wuxing | 0.591 | 0.645 | 0.671 | Longquan | 0.486 | 0.276 | 0.372 |
| Nanxun | 0.386 | 0.5 | 0.57 |  |  |  |  |
| Deqing | 0.439 | 0.551 | 0.561 |  |  |  |  |
| Changxing | 0.465 | 0.521 | 0.59 |  |  |  |  |
| Anji | 0.417 | 0.527 | 0.556 |  |  |  |  |
| Yuecheng | 0.616 | 0.566 | 0.644 |  |  |  |  |
| Keqiao | 0.599 | 0.615 | 0.731 |  |  |  |  |
| Shangyu | 0.504 | 0.587 | 0.641 |  |  |  |  |
| Xinchang | 0.365 | 0.53 | 0.455 |  |  |  |  |

Table5: Result of univariate necessity analysis of Mountainous/Island

| Subsystem | 2020 year | | | | 2021year | | | | 2022year | | | |
| --- | --- | --- | --- | --- | --- | --- | --- | --- | --- | --- | --- | --- |
|  | High CCD | | ~High CCD | | High CCD | | ~High CCD | | High CCD | | ~High CCD | |
|  | Consistency | Coverage | Consistency | Coverage | Consistency | Coverage | Consistency | Coverage | Consistency | Coverage | Consistency | Coverage |
| Economic Aggregate (Z1) | 0.609 | 0.270 | 0.412 | 0.866 | 0.310 | 0.800 | 0.044 | 0.800 | 0.609 | 0.270 | 0.412 | 0.866 |
| ~ Economic Aggregate (Z1) | 0.699 | 0.200 | 0.653 | 0.888 | 0.923 | 0.120 | **0.989** | **0.910** | 0.699 | 0.200 | 0.653 | 0.888 |
| Social Security (Z5) | 0.775 | 0.227 | 0.587 | 0.815 | 0.168 | 0.598 | 0.040 | 1.000 | 0.775 | 0.227 | 0.587 | 0.815 |
| ~ Social Security (Z5) | 0.368 | 0.158 | 0.443 | 0.903 | 1.000 | 0.128 | **0.984** | **0.893** | 0.368 | 0.158 | 0.443 | 0.903 |
| Social Assistance (Z6) | 0.678 | 0.327 | 0.312 | 0.715 | 0.787 | 0.200 | 0.500 | 0.897 | 0.678 | 0.327 | 0.312 | 0.715 |
| ~ Social Assistance (Z6) | 0.409 | 0.111 | 0.706 | 0.912 | 0.594 | 0.144 | 0.554 | 0.948 | 0.409 | 0.111 | 0.706 | 0.912 |
| Healthcare Service Accessibility (Z7) | 0.485 | 0.199 | 0.470 | 0.917 | 0.661 | 0.472 | 0.145 | 0.733 | 0.485 | 0.199 | 0.470 | 0.917 |
| ~ Healthcare Service Accessibility (Z7) | 0.798 | 0.241 | 0.589 | 0.845 | 0.626 | 0.094 | **0.895** | **0.949** | 0.798 | 0.241 | 0.589 | 0.845 |
| Healthcare Service Fairness (Z8) | 0.828 | 0.298 | 0.471 | 0.803 | 0.939 | 0.321 | 0.332 | 0.800 | 0.828 | 0.298 | 0.471 | 0.803 |
| ~ Healthcare Service Fairness (Z8) | 0.453 | 0.153 | 0.588 | 0.942 | 0.416 | 0.081 | 0.719 | 0.988 | 0.453 | 0.153 | 0.588 | 0.942 |

Table6: Analysis of sufficient conditions at consistency > 0.80 and frequency = 1 (Intermediate solution).

| Configuration | 2020 year | | | | | | 2021 year | | | 2022year | | |
| --- | --- | --- | --- | --- | --- | --- | --- | --- | --- | --- | --- | --- |
|  | high CCD | ~high CCD | | | | | high CCD | ~high CCD | | high CCD | ~high CCD | |
|  | 1 | 1a | 2a | 3a | 4a | 5a | 1 | 1b | 2b | 1 | 1c | 2c |
| Economic Aggregate (Z1) | ⊗ | ⊗ |  | • |  | ⊗ | ⊗ | ⊗ | ⊗ | ⊗ | ⊗ | ⊗ |
| Social Security (Z5) | ● |  | • | • | • | ⊗ | ⊗ | ⊗ | ⊗ | ⊗ | ⊗ | ⊗ |
| Social Assistance (Z6) | ● | **⊗** | **⊗** |  |  | **⊗** | • |  | • | ● |  | **⊗** |
| Healthcare Service Accessibility (Z7) | **⊗** |  |  | ● | ● | ⊗ | ● | **⊗** |  | ● | **⊗** |  |
| Healthcare Service Fairness (Z8) | • | ⊗ | ⊗ |  | • |  | ● |  | **⊗** | • |  | • |
| consistency | 0.828 | 0.998 | 0.907 | 0.891 | 0.875 | 1.000 | 0.701 | 0.952 | 1.000 | 0.735 | 0.946 | 0.998 |
| raw coverage | 0.255 | 0.415 | 0.275 | 0.334 | 0.297 | 0.314 | 0.545 | 0.889 | 0.344 | 0.519 | 0.908 | 0.393 |
| unique coverage | 0.255 | 0.017 | 0.043 | 0.043 | 0.032 | 0.044 | 0.545 | 0.584 | 0.039 | 0.519 | 0.563 | 0.048 |
| solution consistency | 0.828 | 0.918 | | | | | 0.701 | 0.954 | | 0.735 | 0.948 | |
| solution coverage | 0.255 | 0.795 | | | | | 0.545 | 0.928 | | 0.519 | 0.956 | |

Note: Black circles(•)indicate the presence of a condition, and circles with“x”( ⊗)indicate its negation. Large circles indicate core conditions, and small ones represent peripheral

Table7: Robustness check summary

| Solution | 2020 year | | | | | | 2021 year | | | 2022year | | |
| --- | --- | --- | --- | --- | --- | --- | --- | --- | --- | --- | --- | --- |
|  | high CCD | ~high CCD | | | | | high CCD | ~high CCD | | high CCD | ~high CCD | |
|  | 1 | 1a | 2a | 3a | 4a | 5a | 1 | 1b | 2b | 1 | 1c | 2c |
| Consistency≧0.75 | ✓ | ✓ | ✓ | ✓ | ✓ | ✓ | ✓ | ✓ | ✓ | ✓ | ✓ | ✓ |
| Consistency≧0.80 | ✓ | ✓ | ✓ | ✓ | ✓ | ✓ | ✓ | ✓ | ✓ | ✓ | ✓ | ✓ |
| Consistency≧0.85 | ✓ | ✓ | ✓ | ✓ | ✓ | ✓ | ✓ | ✓ | ✓ | ✓ | ✓ | ✓ |

Note: ✓ is placed in cases where the same solution paths occurs. (✓) is placed where the solution term is comparable, but 1 condition is added or removed vs. initial analysis

Table8:The cases of each Configurations(Supplementary)

| year | Configuration | path | Case | typical case |
| --- | --- | --- | --- | --- |
| 2020 | Social Security-Social Assistance Dual Driver Type | 1 | 2020-73 (0.56,0.8) | 2020-73 |
|  | Social Assistance Constraint Type | 1a | 2020-28 (0.99,1), 2020-52 (0.95,0.99), 2020-26 (0.94,0.92), 2020-84 (0.94,1), 2020-63 (0.88,0.99), 2020-24 (0.75,0.82), 2020-23 (0.66,1), | 2020-28 |
|  |  | 2a | 2020-64 (0.98,0.9), 2020-86 (0.97,0.59), 2020-84 (0.84,1), 2020-23 (0.81,1),  2020-24 (0.75,0.82), 2020-79 (0.55,0.43) |  |
|  |  | 5a | 2020-28 (0.99,1), 2020-52 (0.99,0.99), 2020-63 (0.97,0.99), 2020-85 (0.88,1), 2020-26 (0.8,0.92), 2020-27 (0.51,1) |  |
|  | High Healthcare Service Accessibility Constraint Type | 3a | 2020-8 (0.99,0.99), 2020-68 (0.99,1), 2020-72 (0.93,0.2), 2020-81 (0.9,1),  2020-61 (0.85,1), 2020-60 (0.57,0.99), 2020-86 (0.51,0.59) | 2020-8 |
|  |  | 4a | 2020-8 (1,0.99), 2020-81 (1,1), 2020-61 (0.97,1), 2020-72 (0.96,0.2),  2020-62 (0.66,1), 2020-60 (0.57,0.99), 2020-82 (0.54,1) |  |
| 2021 | Healthcare Service Accessibility-Healthcare Service Fairness Dual Driver Type | 1 | 2021-26 (0.79,0.85) | 2021-26 |
|  | Healthcare Service Accessibility Constraint Type | 1b | 2021-27 (0.99,1), 2021-84 (0.99,1), 2021-85 (0.99,1), 2021-86 (0.99,1),  2021-54 (0.98,1), 2021-81 (0.98,0.95), 2021-82 (0.96,0.97), 2021-28 (0.95,1), 2021-60 (0.95,0.97), 2021-23 (0.94,0.99), 2021-61 (0.94,0.98), 2021-62 (0.94,1), 2021-72 (0.93,0.98), 2021-63 (0.91,0.82), 2021-74 (0.91,0.96), 2021-79 (0.9,0.95), 2021-80 (0.9,0.85), 2021-73 (0.89,0.61), 2021-83 (0.83,0.99), 2021-64 (0.67,0.97), 2021-52 (0.61,0.95), 2021-24 (0.55,0.07), 2021-8 (0.51,0.91) | 2021-27 |
|  | Healthcare Service Fairness Constraint Type | 2b | 2021-28 (0.96,1), 2021-27 (0.77,1), 2021-72 (0.76,0.98), 2021-68 (0.75,0.83), 2021-52 (0.74,0.95), 2021-79 (0.68,0.95), 2021-61 (0.64,0.98) | 2021-28 |
| 2022 | Social Assistance-Healthcare Service Accessibility Dual Driver Type | 1 | 2022-26 (0.83,0.85) | 2022-26 |
|  | Healthcare Service Accessibility Constraint Type | 1c | 2022-23 (0.99,0.99), 2022-27 (0.99,1), 2022-28 (0.99,1), 2022-62 (0.99,1), 2022-68 (0.99,0.83), 2022-85 (0.99,1), 2022-54 (0.98,1), 2022-61 (0.98,0.98), 2022-84 (0.98,1), 2022-60 (0.97,0.97), 2022-74 (0.96,0.96), 2022-86 (0.96,1), 2022-63 (0.95,0.82), 2022-72 (0.94,0.98), 2022-73 (0.94,0.61), 2022-79 (0.94,0.95), 2022-81 (0.94,0.95), 2022-82 (0.9,0.97), 2022-64 (0.88,0.97), 2022-83 (0.76,0.99), 2022-80 (0.65,0.85), 2022-24 (0.56,0.07), 2022-8 (0.54,0.91) | 2022-27 |
|  | Social Assistance Constraint Type | 2c | 2022-54 (0.99,1), 2022-60 (0.97,0.97), 2022-82 (0.95,0.97), 2022-52 (0.87,0.95), 2022-80 (0.87,0.85), 2022-81 (0.87,0.95), 2022-63 (0.8,0.82) | 2022-54 |

Note:2020, 2021, and 2022 represent the years, and the number following each year indicates the number of countries (see Table 9 for details)

Table9:The number of all counties(Supplementary)

| number | name | number | name |
| --- | --- | --- | --- |
| 1 | Xiaoshan | 44 | Yuecheng |
| 2 | Yuhang | 45 | Keqiao |
| 3 | Linping | 46 | Shangyu |
| 4 | Qiantang | 47 | Xinchang |
| 5 | Fuyang | 48 | Zhuji |
| 6 | Linan | 49 | Shengzhou |
| 7 | Tonglu | 50 | Wucheng |
| 8 | Chunan | 51 | Jindong |
| 9 | Jiande | 52 | Wuyi |
| 10 | Haishu | 53 | Pujiang |
| 11 | Jiangbei | 54 | Panan |
| 12 | Beilun | 55 | Lanxi |
| 13 | Zhenhai | 56 | Yiwu |
| 14 | Yinzhou | 57 | Dongyang |
| 15 | Fenghua | 58 | Yongkang |
| 16 | Xiangshan | 59 | Kecheng |
| 17 | Ninghai | 60 | Qujiang |
| 18 | Yuyao | 61 | Changshan |
| 19 | Cixi | 62 | Kaihua |
| 20 | Lucheng | 63 | Longyou |
| 21 | Longwan | 64 | Jiangshan |
| 22 | Ouhai | 65 | Dinghai |
| 23 | Dongtou | 66 | Putuo |
| 24 | Yongjia | 67 | Daishan |
| 25 | Pingyang | 68 | Shengsi |
| 26 | Cangnan | 69 | Jiaojiang |
| 27 | Wencheng | 70 | Huangyan |
| 28 | Taishun | 71 | Luqiao |
| 29 | Ruian | 72 | Sanmen |
| 30 | Yueqing | 73 | Tiantai |
| 31 | Longgang | 74 | Xianju |
| 32 | Nanhu | 75 | Wenling |
| 33 | Xiuzhou | 76 | Linhai |
| 34 | Jiashan | 77 | Yuhuan |
| 35 | Haiyan | 78 | Liandu |
| 36 | Haining | 79 | Qingtian |
| 37 | Pinghu | 80 | Jinyun |
| 38 | Tongxiang | 81 | Suichang |
| 39 | Wuxing | 82 | Songyang |
| 40 | Nanxun | 83 | Yunhe |
| 41 | Deqing | 84 | Qingyuan |
| 42 | Changxing | 85 | Jingning |
| 43 | Anji | 86 | Longquan |
